# Generating a consensus on an undergraduate curriculum for paediatric caries management: a protocol for a Delphi and nominal groups study

**DOI:** 10.1186/s12909-025-06985-3

**Published:** 2025-07-01

**Authors:** Helen J. Rogers, Faith Campbell, Nicola Innes

**Affiliations:** 1 School of Dental Sciences, Faculty of Medical Sciences, Newcastle University, Newcastle upon Tyne, UK; 2 School of Dentistry, Park Place, University of Dundee, Dundee, UK; 3 Glasgow Dental Hospital and School, 378 Sauchiehall St, Glasgow, UK; 4 School of Dentistry, Dental Drive, Heath Park, College of Biomedical and Life Sciences, Cardiff University, Cardiff, UK

**Keywords:** Caries, Curriculum development, Consensus, Delphi, Dental

## Abstract

**Background:**

Untreated dental caries is the most common condition to affect children globally, and significantly affects their oral health-related quality of life. Our understanding of caries and its management has progressed significantly over the past two decades, though a number of Dental Schools in the United Kingdom (UK) continue to teach traditional techniques, which no longer align with the evidence base. There is a clear need for an evidence-based curriculum for paediatric caries management for UK Dental Schools. This protocol details a study to generate consensus on what should be included in such a curriculum.

**Methods:**

Preliminary research by the authors will be used to identify techniques to be considered for inclusion in the curriculum and their definitions will be drawn from scientific literature. A total of 21 national and international experts in paediatric cariology will be invited to take part in a Delphi survey, via the Welphi application. Participants will be given a list of techniques, with associated definitions, and asked to state whether each should be included in the curriculum, or not, or if they are unsure. Techniques with at least 70% agreement will be removed from future survey rounds. Participants will be provided with feedback regarding all techniques not reaching a consensus, and asked to re-evaluate these again, up to a maximum of four rounds. Seven UK-based Delphi participants will then be invited to participate in a Nominal Group Technique approach, to confirm suitability of the techniques agreed for inclusion in a UK curriculum, reviewing and discussing where agreement was previously not reached. The final list of approved techniques will inform a UK paediatric caries management curriculum, which will be developed with involvement of stakeholders. Endorsement for this curriculum will be sought from the British Society of Paediatric Dentistry. The UK General Dental Council will be asked to consider its content as part of the standards for UK Dental Schools. Following dissemination of the curriculum we will seek to investigate whether there is greater alignment across UK Dental Schools with the evidence base for cariology.

**Conclusion:**

This study proposes a rigorous approach to curriculum development with active involvement of a broad range of stakeholders. These methods can be applied to development of further curricula in dental education, benefitting the teaching community, students and patients.

## Background

Untreated dental caries is the most common condition to affect children globally, causing pain, difficulty eating and sleeping [1]. Our understanding of caries, and its management, has developed significantly over the past 20 years [2]. There is now an overwhelming body of evidence to demonstrate that caries can arrest when isolated from the plaque biofilm, through measures that include effectively ‘sealing’ caries into the tooth, reducing the need for invasive operative techniques. These aptly named biological (as opposed to surgical) approaches are particularly applicable in paediatric dentistry, and are endorsed by a range of guidelines and organisations [3–5]. One such approach is the Hall Technique for management of carious primary molars, whereby a preformed metal crown is cemented over the tooth, without the need for local anaesthetic, caries removal, or prior or reduction of the tooth surface, with a strong body of evidence demonstrating a 97% success rate at 5 years [6]. Another contemporary approach involves placement of a resin fissure sealant over occlusal caries in permanent molars, without prior caries removal, whereby the evidence suggests that 90% of lesions managed this way will not progress. This avoidance of tooth tissue removal can prevent or delay the commencement of the restorative cycle, enabling a tooth to be preserved for longer, which is of particular importance for children and young people [7]. 

The General Dental Council (GDC) is the regulatory body for the dental profession in the United Kingdom (UK), and sets a standard for graduating dental professionals to meet [8]. This standard comprises broad learning outcomes, without specifying which treatments students should be able to provide, how the students should be taught about these treatments, or how they should be assessed to demonstrate competence. These are all at the discretion of the individual Dental Schools.

A previous cross-sectional study undertaken by the authors identified not only a lack of consistency in undergraduate paediatric cariology teaching across 14 participating dental schools in the UK [9], but also outdated caries management techniques or methods that would no longer be expected to be provided by a Dentist or Dental Therapist. Furthermore, some schools had not incorporated more modern evidence-based caries management approaches into their teaching. As a result, we can expect a wide variation in the knowledge and skills that newly graduated dentists and therapists will commence their working career with. Moreover, paediatric patients are likely to receive very different care depending on where their dentist has graduated from. Anecdotally, inspections of Dental Schools undertaken by the GDC have continued to request evidence that conventional/outdated techniques are being taught, undertaken and assessed for undergraduate students. Those schools providing modern, evidence-based techniques are being required to provide a robust defence for their teaching. Unfortunately there is no publicly available guidance from the GDC to specify which caries management approaches should be taught, and how these should be assessed.

This slow movement of change to adopt evidence and failure to both wholly embed modern caries management approaches and withdraw outdated techniques from undergraduate dental courses across the UK is representative of the process of adoption of new understandings of science [10]. Knowledge accumulation was once thought to be an incremental, logical process within science. Thomas Kuhn’s more humanistic, episodic model, however, explains the process as consisting of periods of conceptual agreement amongst scientists where knowledge gradually grows through research, is now widely accepted [10]. There then comes a time when anomalies are noted and accumulate. These don’t agree with conventional wisdom and a period of revolutionary science requires the community to re-think their ideas and they are pushed to adopt new ideas and paradigms into their day-to-day understanding. Kuhn argued that this paradigm shift involves a complex process of sociological shift, enthusiasm, as well as confusion and refutation before the new knowledge becomes a part of accepted doctrine. He describes 5 phases: Phase 1. The pre-paradigm phase where there is no consensus on a single theory; Phase 2– Normal science where puzzles can be framed and solved within the dominant paradigm; Phase 3– the paradigm is unable to account for anomalies that arise and there is a period of dissonance; Phase 4– Paradigm shift or scientific revolution is when the assumptions underlying the paradigm are examined and a new one is established that fits better; and Phase 5– Post-revolution, there is a new paradigm that fits and a period of normal science resumes where the puzzles that arise are solved within this frame. It could be considered that Paediatric Dentistry in the UK is somewhere between Phase 3 (crisis in understanding) and entering Phase 4 (the paradigm shift phase).

Although creating a core curriculum is the creation of new evidence in itself, it also facilities the enactment of the evidence, being a framework within which those delivering education will be required to operate. The imperative to disseminate new scientific understanding through new practitioners can be facilitated by ready access to a curriculum specifically for paediatric caries management. An evidence-based, consensus-based curriculum on this topic, followed by all UK dental schools, is one way to help to ensure consistency in teaching and ultimately improve care for children with caries.

The Core Cariology Curriculum proposed jointly by the Organization for Caries Research (ORCA) and Association for Dental Education in Europe (ADEE) provides a clear outline of the learning outcomes expected for undergraduate education in relation to dental caries [11]. Nonetheless, this curriculum does not specify exactly which caries management techniques should be taught to undergraduate students, or how these techniques should be assessed.

The research outlined in this protocol forms a key part of a comprehensive programme of research that is underway to develop, and implement, a UK undergraduate caries management curriculum for paediatric patients. This component of the research aims to gain consensus on the paediatric caries management techniques to be taught to undergraduate dentistry and therapy students prior to graduation at the level of a safe practitioner. This curriculum is intended to complement the Core Cariology Curriculum from ORCA and ADEE within a UK setting, providing clear guidance on the techniques that a new graduate should be expected to provide. The specific objectives are to:

1. identify and define the full range of caries management techniques for paediatric patients for consideration for inclusion in a national paediatric cariology curriculum;
2. identify experts in paediatric cariology within the UK and internationally for consideration for consensus development of a national paediatric cariology curriculum;
3. develop a consensus between experts, on caries management approaches that should be taught and assessed for undergraduate dentistry, dental hygiene and dental therapy students; and
4. refine and confirm the final range of approaches for a UK undergraduate curriculum.

## Methods

Delphi and NGT are widely-used consensus methods, involving expert panellists to determine priorities for important, unsettled issues, such as the topic of the present study [12]. The former is typically undertaken anonymously, online, whilst the latter is undertaken in-person. This study combines these approaches as detailed below.

### Justification for study design

Delphi methods will be used to achieve objective 3. The Delphi method has a number of advantages as a consensus approach, in that it provides participants with anonymity, can easily incorporate participants from across the globe, and ensures that all participants have equal voice, preventing the dominance that some individuals can have in a group setting [13]. Through using this approach initially, the study will benefit from the expertise and experiences of international experts, which is of particular importance given that there are a finite number of UK-based paediatric cariology experts, and heterogeneity within a Delphi panel is considered ideal. The Delphi method has been used in similar studies in other areas of healthcare previously [14, 15]. 

To achieve objective 4, NGT will be used. The NGT uses a highly structured meeting involving reflection, discussion and rating a series of issues [16]. Given the smaller number of experts required for this approach, it will be possible, and is desirable, to undertake this with UK-based experts only. This will ensure that the final list of techniques to be included in the curriculum meets the approval of UK experts, and is applicable for UK undergraduate students and environment. This approach will also provide an opportunity to focus on the issues that did not gain consensus in the Delphi stage of the study. The two approaches have been used together in this way previously, and are sometimes referred to as a Modified Delphi Technique [17]. 

### Identify and define the full range of paediatric caries management techniques

A scoping review was recently conducted by this research group to identify all available guidelines on caries management relevant for paediatric patients published from 2007 onwards, covering a period of significant developments in our understanding of caries [5]. This review was undertaken using the Joanna Briggs Institute systematic methods. A search of electronic databases for peer reviewed literature was performed using Cochrane Library, MEDLINE via PubMed, TRIP Medical Database and Web of Science. Hand searching was undertaken for grey literature. Following quality appraisal using the AGREE II criteria [18], this review identified a total of eight guidelines that were deemed to be of a suitable standard to inform the present study.

The selection process for guidelines suitable for inclusion in the review followed the Appraisal of Guidelines for Research and Evaluation II (AGREE II) tool for quality appraisal of guidelines, which led to a global quality score [18]. Quality appraisal was undertaken independently by at least two reviewers, who then met to discuss any disagreements, with the inclusion of a third reviewer to reach consensus if required [5]. Guideline selection was guided by the minimum score of 4.5 in the overall AGREE II quality scoring system, but reviewers also included wider considerations relating to the relevance to the UK education and wider paediatric dentistry environment as well as the paediatric caries-specific curriculum. In the review, 581 studies were identified for screening, 33 were assessed for full text eligibility, 16 were suitable for inclusion and following quality appraisal, eight were carried forward for analysis [5]. This highlighted the lack of high-quality guidelines available to inform a paediatric cariology curriculum [5]. All techniques mentioned in these guidelines will be put forward to the Delphi panel in the following stage.

As more recent guidelines may exclude some traditional techniques that are still being provided by some dental schools, this approach will be supplemented by techniques captured by the aforementioned cross-sectional study undertaken by this research group [9]. 

Definitions for all techniques will be derived from the ORCA and Cariology Research Group of the International Association for Dental Research consensus report on the terminology of dental caries and dental caries management [19]. Definitions for any techniques excluded from this consensus report will be identified from the guidelines and wider literature if necessary. All definitions will be reviewed by the research steering group to ensure their comprehension within a UK setting, and any necessary modifications will be made. Further information on the research steering group is discussed in the ‘Project Management’ section of this article.

### Selection of the Delphi panel

Each member of the steering group will nominate seven renowned cariology experts who will be invited to join the Delphi panel, culminating in a total of 21 panellists, with anticipation of attrition rates up to 17%.^13^ There is a very small field of clinician cariologists who meet our definition of an expert for this study so nominations will be undertaken through both known contacts in their networks and looking up experts in cariology. There is no agreement in the literature regarding the optimal size of a Delphi panel, though in general larger panels are considered to improve the reliability of the outcomes [20]. Within the context of this study, recruitment will be limited by the number of paediatric cariology experts meeting the inclusion criteria (described below), given that Paediatric Dentistry is a relatively small specialty, and there are few clinicians, academics and clinical academics researching this particular field. Furthermore, the composition of the panel involving relevant and interested experts is generally considered to be more of a priority than the size of the panel [21]. There is no a priori minimum number of participants required to proceed, however should participation drop below 17 experts, further expert involvement may be sought.

Experts will be defined as meeting at least one of the following criteria;

- individuals with significant experience of managing caries in children clinically, and/or;
- with at least ten original research publications related to cariology, with experience of obtaining relevant competitive research funding and/or;
- have presented as invited keynote speakers on cariology/paediatric caries management, at international conferences.

Experts unable to complete a survey in English language will be excluded, as translation software cannot be relied upon to accurately capture the details of each technique. Panellists will be selected to incorporate international representation, whilst ensuring heterogenous expertise from different universities across devolved UK nations to ensure the final consensus is appropriate for a UK (National Health Service-focussed) setting. This approach will optimise buy-in from Higher Education Institutions to the new caries curricula developed as an output of this research and maximise potential for future transferability to other countries. Following screening by the Steering Committee to ensure that potential panellists meet the eligibility criteria outlined above, an invitation will be sent to each via a personal email from a member of the research team, with a study information sheet attached. Should no response be obtained within 14 days, a reminder will be sent and if there is still no response within 7 days, an alternative expert will be invited.

### Delphi methods

Panellists will be invited to download Welphi (Welphi© Lisbon, Portugal), an application designed for use with Delphi techniques. They will be asked to provide consent via Welphi and declare any conflicts of interest. Panellists will then be presented with a definition and explanation of each included caries management technique, and will be required to respond to the question ‘Should this technique be included in an undergraduate paediatric caries management curriculum?’ The response format will include three options (‘yes’, ‘no’ and ‘unsure’), rather than using a Likert scale, to minimise the potential for panellists to give a neutral response [22]. In line with previous studies of this type, it is unlikely that a complete consensus will be achieved through Delphi, hence for this stage of the study, consensus will be defined as being at least 75% agreement.^13^

Panellists will remain anonymous during the survey rounds. Following each round, the panellists will be provided with controlled feedback, summarising the analysed results (plus any anonymous comments) in an easily interpretable format through the Welphi application. This informs each panellist about the trend, allowing them to consider their response and change it if they wish. All items where consensus exists will be dropped from future rounds. In line with recommendations on Delphi technique, the number of rounds conducted will not be prespecified; the rounds will continue until no further prioritisation is warranted, with a maximum of four rounds for pragmatic reasons [23]. Any caries management techniques for which consensus cannot be achieved after four successive rounds will be carried forward for discussion within the Nominal Group Technique stage, and the Delphi will be closed. As successive rounds can increase participation fatigue, a response rate of 70% will be accepted as valid for subsequent rounds, in line with previous research [21]. 

Successive iterative rounds of the Delphi will be analysed, allowing for comparison of consensus and stability between each round. This can be undertaken within the Welphi application, and will involve measurement of central tendencies, with dispersion, percentage and frequency of distribution, in line with previous studies [23]. 

Qualitative comments provided by panellists will be used to improve the framing of statements and definitions for subsequent rounds, to increase the potential to reach a stable consensus.

### Nominal group technique (NGT)

The NGT provides a highly structured, face-to-face group interaction, comprising four steps outlined by Delbecq and Van de Ven, detailed below [24]. A maximum of seven UK-based panellists who participated in the Delphi will be invited to take part in the NGT, in line with recommendations in the literature [12]. This process will be guided by an experienced facilitator and held in a meeting room in a convenient location.

*Silent reflection: P*articipants will consider and record their thoughts on the relevance of the techniques that gained consensus for inclusion during the Delphi for a UK curriculum.

#### Round robin

Participants will then be asked, in turn, to share a single reflection on a technique with the group, which will not be discussed at this time, but simply recorded on a flipchart. This will continue until no further reflections are forthcoming.

#### Clarification

The reflections will then be clarified through discussion, whereby similar reflections can be grouped, with agreement from all participants. The facilitator will not provide any direction, though will advise participants that there is no requirement for agreement to be reached at this stage.

#### Voting

The final stage requires participants to vote upon techniques that have been discussed, which may be a subset of contentious techniques drawn from the initial Delphi, including those that did not gain consensus within four rounds, that should be ranked in terms of importance for inclusion in the curriculum. Votes are made on paper, in confidence, prior to being given to the facilitator. The scores for each item are then summed by the facilitator and shared with the group for discussion and decision. If indicated, the voting stage can be repeated to allow participants to revise their ranking following the discussion and feedback.

Each stage of the NGT will be documented clearly through written summaries and audio recordings with field notes, and will inform the subsequent stage. The results of the voting stage will be analysed with simple descriptive statistics.

The final list of approved techniques will form the basis of the new curriculum.

### Curriculum development

The curriculum itself will be developed by the Steering Group with involvement of a broad range of stakeholders, including members of the Advisory Group, which includes children, parents and undergraduate students, alongside invited representatives from the British Society of Paediatric Dentistry (BSPD), which will include those involved in delivering undergraduate teaching, and the GDC. Further details of the study Advisory Group and their role in this study are provided in the Project Management section below.

The curriculum itself will comprise details of the techniques agreed through the previous stages of this study, with clear learning outcomes for each, and recommendations on how these techniques may be taught and assessed. Once developed, it will be sent out to members of the Teachers’ Branch of BSPD for consultation.

Future research is planned using a qualitative approach to inform development of an implementation plan, prior to the release of the new curriculum. Previously described stakeholders will be invited to participate in this aspect. This will ensure that integration with existing curricula within each institution is as straightforward as possible.

### Project management

#### Research steering group

The Research Steering Group for this study comprises three paediatric dentists, all with extensive clinical experience in managing caries in children and knowledge and experience of UK education for dental clinicians. The group have varied levels of research experience, including an internationally renowned professor of paediatric dentistry, a mid-career researcher with a PhD, and a current PhD student. Their specific research interests encompass the clinical management of caries, the quality of life impacts of caries on children, and the oral health of children with multi-morbidity.

The Research Steering Group will make joint decisions regarding the conduct of this study, but will not participate in the Delphi or NGT and will not facilitate the NGT due to the potential for influence over decisions. An independent dentist outside of the specialty of Paediatric Dentistry will instead be invited to act as facilitator.

#### Advisory group

Patients, parents and undergraduate dentistry and therapy students are all important stakeholders in this study, though within the context of gaining a consensus for curriculum development, an in-depth technical knowledge of caries management technique is required. As such, these groups will not be invited as participants in the Delphi and NGT, but will be actively involved in this study in other ways.

Children will ultimately be impacted by the outcomes of the curriculum developed, as they will experience the care provided by those participating in the new curriculum, hence their involvement in the study is key. Patients are increasingly being involved in curriculum development in medical education, with benefits including enhancing patient satisfaction with treatment, improved care quality, improved social accountability of institutions, enhancing empathy and interprofessional working of professionals [25]. The involvement of children and parents will help to enhance the relevance and quality of the research, and the acceptability of the final curriculum to patients and families.

A study Advisory Group will guide decisions made by the Research Steering Group. Six children aged 5 to 16 years with experience of dental caries, and their parents will be invited from existing Patient and Public Involvement and Engagement groups in Newcastle, Cardiff and Dundee to join the Advisory Group, alongside two undergraduate dentistry students and two dental therapy students from each University respectively.

Advisory Group members will be asked to contribute to the development of the curriculum stage in particular, and support the dissemination of study findings. This will involve preparation and delivery of presentations at public engagement events, and development of a supporting statement that will be circulated with the curriculum as it is sent out for consultation.

Advisory Group members can choose to contribute to different aspects of the study and can join meetings remotely to maximise flexibility. Shopping vouchers will be given to members to thank them for their time, and any costs incurred will be reimbursed, in line with NIHR Involve guidance [26, 27]. 

#### Timeline

**Fig. 1:**
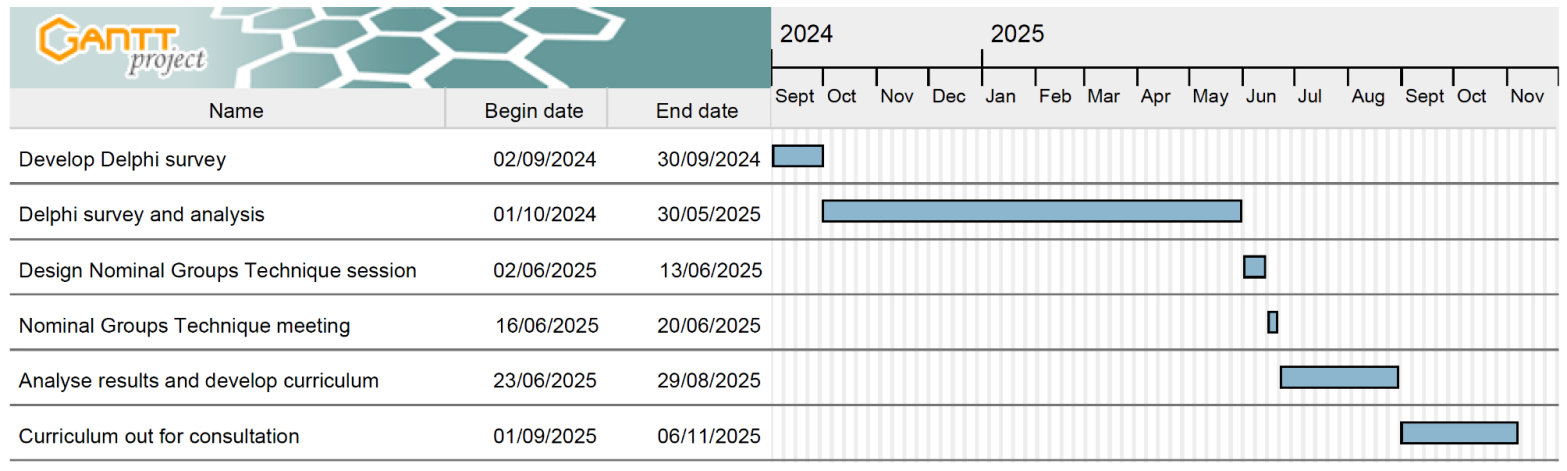
Gantt chart outlining the proposed timeline for this study

The Delphi component of this study is due to commence in September 2024 and will run for a nine-month period. The NGT will be held in June 2025, and the curriculum will be developed during the following two months. It will be circulated for consultation in September 2025 and shared with the BSPD Teachers’ Branch at their annual study day. Further detail can be seen in Fig. 1.

## Discussion

There is a clear need for a UK paediatric caries curriculum, and the use of consensus methodology in this way is appropriate to gain clarity on which techniques should be included. The use of Delphi and NGT in curriculum development is novel within the field of dentistry, with previous dental curricula having been developed using less rigorous approaches, such as expert consensus [28]. Delphi and NGT have been used successfully in studies within other healthcare fields, focussed on curriculum development, and identification of techniques for training purposes [29–31]. A further strength of the present study is the involvement of children, parents and undergraduate students [32, 33]. These groups have a wealth of insight to offer, and their involvement at this early stage could facilitate a smooth implementation of the curriculum.

This research will result in an explicit, evidence-based curriculum supported through consensus and endorsed by the British Society of Paediatric Dentistry. This means that Higher Education Institutions with a role in training dental and dental therapy students will be able to follow the recommendations on content for the undergraduate paediatric cariology curriculum. With effective implementation of more standardised training, there is the potential for all students across the UK to be trained in modern techniques and with up-to-date cariology evidence, which would lead to more consistent treatment planning for patients across the UK. The benefit to patients (children with dental caries that requires treatment) is that they will receive evidence-based care, regardless of where their dental care provider completed their training. This will also reduce the likelihood of children receiving outdated treatments which have been shown to be more invasive, and often unnecessary.

There are a number of practical issues that the steering group will need to consider during this study. Firstly, given that there are a limited number of experts in paediatric cariology that meet our definition, both within the UK and worldwide, there is a risk that participants may experience fatigue [34, 35]. Due to this very small field of paediatric cariologists from whom to select Delphi panellists from, it is inevitable that all panellists will be known to the Research Steering Group. As such, there is the potential for this familiarity of the panellists to influence the results, with their responses being more likely to align with the views and expectations of the Research Steering Group. Poor recruitment and lack of retention of the experts through the Delphi could potentially inhibit progression of the study and invalidate the findings. The authors anticipate that a personalised invitation to participate in the study, sent from a known contact may aid initial recruitment. Also, the use of the novel purpose-built Welphi application may help to improve the user experience of completing the Delphi surveys, and the intrinsic motivation from contributing to a much-needed paediatric caries curriculum may aid retention. Furthermore, the ability to send automated reminders to participants through the application and provide visual and personalised feedback between Delphi rounds may help to prevent attrition.

## Conclusion

In conclusion, this study protocol uses a combination of consensus methods to gain agreement on which paediatric caries management techniques should be included in a much-needed curriculum for UK undergraduate dentistry and therapy students’ programmes. This will directly inform the development of such a curriculum, the availability of which may encourage adoption of modern cariology, restorative and paediatric dentistry techniques, and reduce variation in teaching for programmes in UK Dental Schools. Furthermore, the combination of methodologies used in this study may aid consensus development for other dental curricula, both within the UK and further afield.

## Data Availability

No datasets were generated or analysed during the current study.

## Abbreviations

UK: United Kingdom
NGT: Nominal Group Technique
GDC: General Dental Council
BSPD: British Society of Paediatric Dentistry
